# Effect of IAA on *in vitro* growth and colonization of *Nostoc* in plant roots

**DOI:** 10.3389/fpls.2015.00046

**Published:** 2015-02-05

**Authors:** Anwar Hussain, Syed T. Shah, Hazir Rahman, Muhammad Irshad, Amjad Iqbal

**Affiliations:** ^1^Department of Botany, University College of Science Shankar Campus, Abdul Wali Khan University Mardan, MardanPakistan; ^2^Nuclear Institute for Food and Agriculture, Tarnab PeshawarPakistan; ^3^Department of Microbiology, Kohat University of Science and Technology, KohatPakistan; ^4^Department of Food Science, University College of Science Shankar Campus, Abdul Wali Khan University Mardan, MardanPakistan

**Keywords:** *Nostoc*, putative *ipdC*, endophyte, IAA, phytostimulation, knockout

## Abstract

*Nostoc* is widely known for its ability to fix atmospheric nitrogen and the establishment of symbiotic relationship with a wide range of plants from various taxonomic groups. Several strains of *Nostoc* produce phytohormones that promote growth of its plant partners. *Nostoc* OS-1 was therefore selected for study because of the presence of putative *ipdC* gene that encodes a key enzyme to produce Indole-3-acetic acid (IAA). The results indicated that both cellular and released IAA was found high with increasing incubation time and reached to a peak value (i.e., 21 pmol mg^-1^ch-*a*) on the third week as determined by UPLC-ESI-MS/MS. Also the *Nostoc* OS-1 strain efficiently colonized the roots and promoted the growth of rice as well as wheat under axenic conditions and induced *ipdC* gene that suggested the possible involvement of IAA in these phenotypes. To confirm the impact of IAA on root colonization efficiency and plant promoting phenotypes of *Nostoc* OS-1, an *ipdC* knockout mutant was generated by homologous recombinant method. The amount of releasing IAA, *in vitro* growth, root colonization, and plant promoting efficiency of the *ipdC* knockout mutant was observed significantly lower than wild type strain under axenic conditions. Importantly, these phenotypes were restored to wild-type levels when the *ipdC* knockout mutant was complemented with wild type *ipdC* gene. These results together suggested that *ipdC* and/or synthesized IAA of *Nostoc* OS-1 is required for its efficient root colonization and plant promoting activity.

## INTRODUCTION

Co-existence has evolved in several forms of living organisms for successful life on the planet earth. Cyanobacteria are a group of the most ancient oxygenic phototrophic organisms that served the sole source of oxygen in the earth’s atmosphere about 2.5 billion years back (Buick, 2008). *Nostoc* is a genus of nitrogen fixing cyanobacteria found in a number of environmental niches. Colonies of *Nostoc* are composed of filaments of moniliform cells embedded in a gelatinous sheath. Members of this genus associate symbiotically with diverse taxonomic groups of organisms (such as algae, Bryophytes, Pteridophytes, gymnosperms and angiosperms), benefiting their hosts with nitrogen in the form of nitrates (Rai and Bergman, 2000). In nature, *Nostoc* may grow as epiphytes (Myllys et al., 2007), endophytes or symbionts, making beneficial association (Pankratova et al., 2008; Olsson et al., 2012). In addition to nitrogen and carbon fixation (Adams and Duggan, 2012), symbiotic *Nostoc* can also release phytohormones Indole-3-acetic acid (IAA and cytokinins), which improve plant vigor (Hussain et al., 2010).

Production of IAA by *Nostoc* (Sergeeva et al., 2002) and its involvement in plant-microbe signaling has been known for years (Berg, 2009). Microbes are believed to reduce cell wall integrity of the host cell, colonize there and induce plant roots to release more sugars (Boopathi et al., 2013). Aditionally, IAA may de-repress auxin signaling in plants, helping the bacteria to evade the plant defense system (Zhang et al., 2007). Inhibition of IAA transport or mutation in auxin biosynthesis gene on the other hand reduces root colonization (Karabaghli-Degron et al., 1998; Bossuyt et al., 2012). However, the role of this hormone in root colonization by cyanobacteria is less obvious and has not been determined.

Indole-3-acetic acid can be synthesized by both tryptophan dependant and tryptophan independent pathways, but the former is a usual one while the latter is bioenergetically costly (Prinsen et al., 1993; Akashi and Gojobori, 2002). Prokaryotes biosynthesize IAA from tryptophan through one of the three major pathways, out of the three, plant friendly bacteria can synthesize it via the intermediate, indole-3-pyruvate (Bruto et al., 2014). The first step in the indolepyruvate pathway is the transfer of the amino group from tryptophan to α-ketoglutarate by an enzyme aminotransferase, yielding indole-3-pyruvate and L-glutamate. Indole-3-pyruvate is then converted to indole-3-acetaldehyde by a rate limiting enzyme, indolepyruvate decarboxylase (IPDC; Patten et al., 2013). This enzyme belongs to a family of thiamine diphosphate-dependent decarboxylases which may catalyze degradation (indolepyruvate and phenylpyruvate decarboxylases), production of alcohols (pyruvate decarboxylases) or amino acid synthesis (acetolactate synthases; Schütz et al., 2003).

The biological role of IPDC has been confirmed in several bacteria, including *Enterobacter cloacae* (Koga et al., 1991), *Azospirillum brasilense* (Costacurta et al., 1994) and *Pantoeaag glomerans* (Brandl and Lindow, 1997). This enzyme is encoded by *ipdC* gene reported in the above mentioned and several other bacteria. Whereas, in cyanobacteria, no *ipdC* gene has been experimentally characterized till date but a homolog of *ipdC* known as thiamine pyrophosphate dependent decarboxylase has been found in the genome of *Nostoc PCC 73102* (Sergeeva et al., 2002). The idea that cyanobacteria can synthesize IAA via indolepyruvate pathway was supported by the findings of Sergeeva et al. (2002). Their results demonstrated that various strains of *Nostoc* produced high levels of IAA, when grown on tryptophan rich media. In the current study, we focused on the ability of endophytic *Nostoc* to colonize rice and wheat plants under laboratory conditions. Putative IAA biosynthesis gene *ipdC* was knocked out form *Nostoc* to investigate its impact on root colonization potential of the *Nostoc*.

## METHODOLOGY

### ISOLATION OF ENDOPHYTIC *Nostoc* STRAINS

Wheat and rice plants were uprooted in the month of April and August, respectively, and brought to the laboratory in polythene bags. Soil particles were washed from the root segments (0.5 cm in length) of the selected plants with running water. The root segments were surface sterilized by sequential dipping in 70% (v/v) ethanol for 5 min, and sodium hypochlorite solution (0⋅9%, w/v) for 20 min. The sterilized root samples were again rinsed three times with sterile distilled water to remove traces of sodium hypochlorite and were injured with sterile blade. The root segments were then plated on BG 11 medium (Barker and Tagu, 2000) containing cyclohexamide (400 mg L^-1^) for 3 weeks at 25°C and photoperiod of 8 h light and 16 h dark. These culture conditions were used throughout this report, unless otherwise mentioned.

After 3 weeks of incubation, the endophytic cyanobacterial colonies were picked from the mixed cultures and enriched in liquid BG11 media (20 mL) to obtain a sufficient biomass. Biomass were harvested from the cultures by centrifugation (12000 rpm for 2 min) and re-suspended in 50 mL BG11 medium, vortexed, and spread on plates of BG11 containing cyclohexamide (400 mg L^-1^). The purity of the colonies was checked by light microscopy and pure colonies were transferred to new BG11 plates incubated under the conditions mentioned above.

To validate the procedure of surface sterilization: (i) surface sterilized root segments were soaked and stirred in 5 mL dH_2_O for 1 min. Two hundred microliter suspension was then plated on BG11 agar plates and microbial growth was checked on plates incubated at 25°C under conditions mentioned earlier; (ii) the isolated cyanobacterial cultures subjected to the same procedure of surface sterilization, were inoculated on BG11 medium at 25°C for 3 weeks and recorded for cyanobacterial vitality.

### SCREENING OF *Nostoc* STRAINS FOR IAA

Method of Hussain et al. (2010) was followed to screen different *Nostoc* cultures for IAA production. Simply, the strains were grown for 3 weeks in liquid BG11 media as mentioned earlier with the addition that 1 mL of the supernatent from cyanobacterial culture was mixed with 2 mL Salkowski reagent. The mixture was allowed to stand for 30 min in dark at room temperature and the absorbance were then measured at 540 nm using PerkinElmer Lambda 25 spectrophotometer. The experiment was repeated three times with three replicates for each isolate.

### IDENTIFICATION OF THE SELECTED STRAIN

To identify the selected strain, genomic DNA was isolated from the cultures grown for 15 days by UltraClean Soil DNA isolation Kit TM (Mo Bio Laboratories, Carlsbad, CA, USA) following the manufacturer’s instructions. The 16S rDNA and its adjacent ITS region were PCR amplified using the generic primer set pA (Edwards et al., 1989) and B23S (Lepère et al., 2000) as described in Rajaniemi et al. (2005). Gel purification of the amplified fragment was performed by using Aquapure genomic DNA extraction kit (Bio-Rad, Hercules, CA, USA), which was then cloned in pGEMT vector. After overnight incubation at 4°C, plasmids were transformed into maximum efficiency competent cells (Life Technologies, Rockville, MD, USA) according to the manufacturer’s instruction and plated onto LB Amp (60 μg mL^-1^) agar and incubated overnight at 37°C. Sequencing was done by using ABI PRISM-3100 Genetic Analyzer (Applied Biosystems, Foster City, CA, USA) as described by Webb and Maas (2002).

### GENERATION OF *ipdC* KNOCKOUT MUTANTS IN *Nostoc*

The putative *ipdC* gene was knocked out as described by Hussain et al. (2013). Genomic DNA was isolated from the cultures pre-incubated for 15 days by using UltraClean Soil DNA isolation Kit TM (Mo Bio Laboratories, Carlsbad, CA, USA) and following the instructions of the manufacturers. The putative *ipdC* gene was knocked out from the genome of *Nostoc* by replacing part of the gene sequence with a DNA fragment comprising of the sequence coding for kanamycin resistance (Homologous recombination). Open reading frame of putative *ipdC* was PCR amplified from genomic DNA of *Nostoc* with *IPDC*-F 5′-AACGGCAAAAGTCACATTCC-3′ and *IPDC*-R 5′-TGAGTTGACGAGGCAGTACG-3′. The amplified fragment was then ligated into the SmaI site of pUC19. A 169-bp fragment was dissected from the gene with PsiI and ScaI, and replaced by a kanamycin resistance sequence conserved in pKRP11 by digestion with SmaI (Kaczmarzyk and Fulda, 2010). The construct was then used to transform *Nostoc* cells, as described by Summers et al. (1995). The suspension containing cells/DNA mixture was then plated on BG 11 medium (solidified with agarose) for the preliminary selection of transformants. After 24 h of incubation, kanamycin resistance of the transformants was tested by adding 0.45 mg of kanamycin into the wells made in the agar plates. Homozygous mutant were obtained by successive streaking of the transformant on BG 11 plates supplemented with kanamycin. Complete segregation of the wild alleles and correct incorporation of the knockout cassette was validated by confirmatory PCR reactions. Primers used for complete segregation and validation of replacement of *ipdC* were: forward primer 5′-CTTCGGCACCAATTACGCAG-3′ and reverse primer TTGCCCAACGAAAGTAGCTC-3′. The correct insertion of the kanamycin resistance cassette was confirmed by following Kaczmarzyk and Fulda (2010).

For genetic complementation of the putative *ipdC* mutant *Nostoc*, a modified version of the above mentioned knockout construct was used. In the modified construct, the kanamycin cassette was replaced by chloramphenicol resistance cassette which was released from the vector pKRP10 (Reece and Phillips, 1995). Fragments of *Nostoc* DNA, flanking the chloramphenicol cassette, served as borders for homologous recombination. EagI restriction site was introduced in flanking region at a distance 79 bp from chloramphenicol cassette by changing 3 bp (QuikChange II site-directed mutagenesis kit, Stratagene) using 5′-GCTTTCTGCTTTAAGTCAACGGCCGCAG-3′and 5′ CTGCGGCCGTTGACTTAAAGCAGAAAGC-3′). A 7433 bp fragment, including the open reading frame of putative *ipdC* gene of *Nostoc* with promoter and terminator, was amplified by PCR with forward and reverse primers (5′-GTAGCCCTGCGATCGCTATT-3′and 5′-GATAGGGGCACCAGCAAAGT-3′, respectively), harboring EagI restriction sites to both ends by using Expand Long Template PCR System (Roche Diagnostics) following the instructions of the manufacturer. The PCR amplified DNA fragment was, digested, cloned into the newly created EagI restriction site of the complementation vector and the resulting plasmid was used to transform *Nostoc ipdC* knockout mutant. Initial selection of transformants was made by spreading the DNA/cells mixture on solid BG 11 medium. Three evenly distributed wells were punched into the agar after 24 h to which Cm (0.45 mg) was added. Homozygous *ipdC* complemented *Nostoc* strains were obtained by successive streaking on BG 11 plates containing the antibiotic.

### DETERMINATION OF IAA IN *Nostoc* CULTURES

Extraction and determination of IAA were performed as described by Hussain et al. (2010). Wild type, *ipdC* knockout mutant and *ipdC* complemented strains of *Nostoc* were grown separately in 250 mL liquid BG11 media containing 500 μg mL^-1^ of tryptophan at 25^o^C under a photoperiod of 8 h light (18 l mol photons m^-2^ s^-1^) and 16 h dark for 3 weeks (inoculum density was adjusted to 0.2 μg mL^-1^ ch-*a*), respectively. Cellular as well as released IAA was measured in the cultures on a weekly basis for 3 weeks. For the extraction of IAA, cultures were harvested by centrifugation (16000 rpm for 5 min) and the harvested supernatant was then filtered through nylon filters (22 μm pore size). Bieleski buffer (60% methanol, 25% CHCl_3_, 10% HCOOH, and 5% H_2_O) containing 10 pmol [2H5] IAA as internal standard was used for the extraction of IAA separately from biomass and culture supernatant. After evaporation to dryness, the pooled extract was reconstituted in 5 mL of acidified water (pH 3) and was purified by solid phase extraction column (CHROMA-BOND^®^ HR-XC, 3 ml, 200 mg) following the manufacturer’s instructions. The eluent was dried, re-dissolved in15 mM ammonium formate (pH 4), and analyzed *via* UPLC-ESI–MS/MS under the conditions described by Hussain et al. (2010).

### ASSOCIATION OF *Nostoc* STRAINS WITH PLANT ROOTS

Seeds of *Triticum aestivum* c.v. Uqab 2000 and *Oriza sativa* c.v. Basmati were surface-sterilized by shaking the seeds in 0.1% HgCl_2_ solution for 5 min. After draining out HgCl_2_ solution, the seeds were rinsed five times with sterile distilled water. Surface-sterilized seeds were then grown in Petri plates containing 7 mL half strength nutrient solution under axenic conditions. After germination, 2 mL cyanobacterial suspension (adjusted to an inoculum density of 1 μg mL^-1^ch-*a*) was added to each plate and incubated for 2-weeks at 22 ± 1°C, 60% relative humidity, 12 h photoperiod, and light intensity adjusted to 180 μmol m^-2^s^-1^. Seedlings were harvested after 2-weeks and the chlorophyll were extracted out of the roots with 80% methanol. Growth of *Nostoc* co-cultivated with rice seedlings was estimated by measuring the chlorophyll-a extracted from wild type or Δ*ipdC Nostoc* cells present in the culture media as well as those associated with plant roots. Chlorophyll-*a* contents were determined by recording the OD of the samples at 660 nm. Each treatment was replicated three times and the experiment was repeated twice.

### CHEMICAL COMPLEMENTATION WITH EXOGENOUS IAA

Effect of exogenous IAA on the *in vitro* and in planta (colonization) growth of *Nostoc* was assessed by supplementing this compound in the culture media used to grow mutant *Nostoc* alone or in association with rice root, under growth conditions previously described. To reconstitute the concentration of IAA in mutant strain (Δ*ipdC*), IAA was supplemented to the culture media used for free grown or root associated *Nostoc* in concentration exactly equal to or twice as the difference in its concentration produced by wild type and mutant strains. Plate counting method was also used to count the number of free grown and root associated *Nostoc* for comparison with Chlorophyll-*a* estimation method. Dilutions of the culture of *Nostoc* and grounded root extracts were plated on agar plate to count the colony forming unit. Growth and colonization was estimated after incubation of 15 days.

### EXPRESSION OF *ipdC* IN PLANT ROOT ASSOCIATED *Nostoc*

Quantitative RT-PCR was used to determine the expression of *ipdC* gene in *Nostoc* associated with rice and wheat root and compared their expression with unassociated strains. Surface sterilized seeds of rice and wheat were grown as described above. After a week of germination, seedlings of each species were shifted to test tubes containing a suspension of *Nostoc* (adjusted to an inoculum density of 1 μg mL^-1^ ch-*a*) and incubated for 1 week under axenic conditions. *Nostoc* samples were collected after every 24 h for 1 week. From the root associated *Nostoc*, mRNA was isolated using UltraClean Plant RNA isolation kit (Mo Bio Laboratories), according to the manufacturer’s instruction. To avoid any DNA or enzymes contamination, the RNA containing samples were treated with DNase I (Promega) followed by phenol-chloroform extraction and ethanol precipitation. Spectrophotometer (Nanodrop Technologies) was used to determine the quantity and quality of the RNA. Total RNA was resuspended in 7 μL of deionized water and used as a template to synthesize cDNA following the protocol for First-strand cDNA synthesis (Fermentas) using random hexamers (Fermentas). The cDNA was used as a template for the expression analysis of *ipdC* gene by qPCR using primers specific to this gene of *Nostoc*. For each time point (means for every 24 h), two replicates from each treatment, i.e., free grown *Nostoc*, a negative control (without cDNA template), and a positive control (using *Nostoc* genomic DNA as a template) were analyzed. For each time point, the *rnpB* (the RNA component of RNase P) was analyzed as a reference gene. The reaction mixture (20 μL) for the qPCR was made by mixing 1 ng of cDNA, 250 nM of each specific primer, 10 μL 2 × SYBR green Supermix with Rox (Bio-Rad Laboratories, Inc.) and sterile water. Conditions of the qPCR were the same as described by Soule et al. (2009).

To confirm that each pair of primers will result in a single PCR product, a melting curve analysis was done. The levels of differential gene expression in extracts from the root-associated *and* free grown *Nostoc* were calculated as ratios from the *rnpB*-normalized real-time amplification data.

### PLANT GROWTH EXPERIMENTS

Effect of different strains of *Nostoc*, wild type Os-1, Δ*ipdC* mutant, and Δ*ipdC*p*ipdC* complemented strains on the growth of rice and wheat was screened under axenic conditions. Surface sterilized seeds, described earlier, were soaked in 10 mL suspension of different strains of *Nostoc* (adjusted to an inoculum density of 1 μg mL^-1^ ch-*a*) separately. *Nostoc* loaded and control seeds (not treated with *Nostoc*) were then grown in pots (three seeds per pot) containing sterilized garden soil under axenic conditions for 2 weeks at 22 ± 1°C, 60% relative humidity, 12 h photoperiod, and light intensity adjusted to 180 μmol m^-2^ s^-1^. After harvesting the seedlings, different growth parameters including shoot length, root length, fresh, and dry weight were recorded. Each treatment was replicated three times and the experiment was repeated three times.

### STATISTICAL ANALYSIS

The data was analyzed statistically by performing one way or three-way analysis of variance (ANOVA) and Duncan multiple range test at *p* = 0.05, using SPSS for windows (Version 20.0; SPSS Inc., Chicago, IL, USA). Two samples Student’s *t*-test was also used to check significance of the data obtained in *Nostoc* growth experiment (Microsoft Excel 2013).

## RESULTS

### SCREENING AND IDENTIFICATION OF THE ISOLATES

Three randomly selected plants each of wheat and rice were uprooted and brought to the lab. Three Petri plates were inoculated with four pieces of roots from each plant. Of 36 root pieces processed separately from rice and wheat, only five and two cyanobacterial isolates were found, which indicated the colonization percentage of rice (13.9%) and wheat (5.5%), respectively, (**Table 1**). Whereas, endophytic *Nostoc* was found only in rice roots. Growth of cyanobacterial colonies started on the 6th day of the incubation of root segments on BG11 agar plates and the majority of the isolates (4 out of 7) were identified as *Nostoc* when their morphology was studied under a light microscope. IAA analysis revealed that *Nostoc* (Os-1) strain was the only one to synthesize IAA among the various *Nostoc* isolates (**Table 1**). The identity of the strain was further confirmed by assessing the homology of the partial sequence of its 16S-23S rDNA intergenic spacer region and the sequence was deposited in NCBI GenBank (accession number KP001508).

**Table 1:** Strains of cyanobacteria isolated from roots of wheat and rice.

Strains	Source	Identification	IAA (colorimetric)	Putative *ipdC* by PCR
Os-1	*Oryza sativa* L.	*Nostoc*	22 ± 1.2	Yes
Os-2	*Oryza sativa* L.	*Phormidium*	1 ± 0.05	No
Os-3	*Oryza sativa* L.	*Nostoc*	BDL	No
Os-4	*Oryza sativa* L.	*Nostoc*	BDL	No
Os-5	*Oryza sativa* L.	*Nostoc*	1.3 ± 0.1	No
Te-1	*Triticumaestivum* L.	*Anabaena*	31 ± 1.7	No
Te-2	*Triticumaestivum* L.	*Anabaena*	17 ± 1.2	No

*BDL, below detection limit.*

### EXPRESSION OF *ipdC* IN ROOTS ASSOCIATED *Nostoc*

**FIGURE 1:**
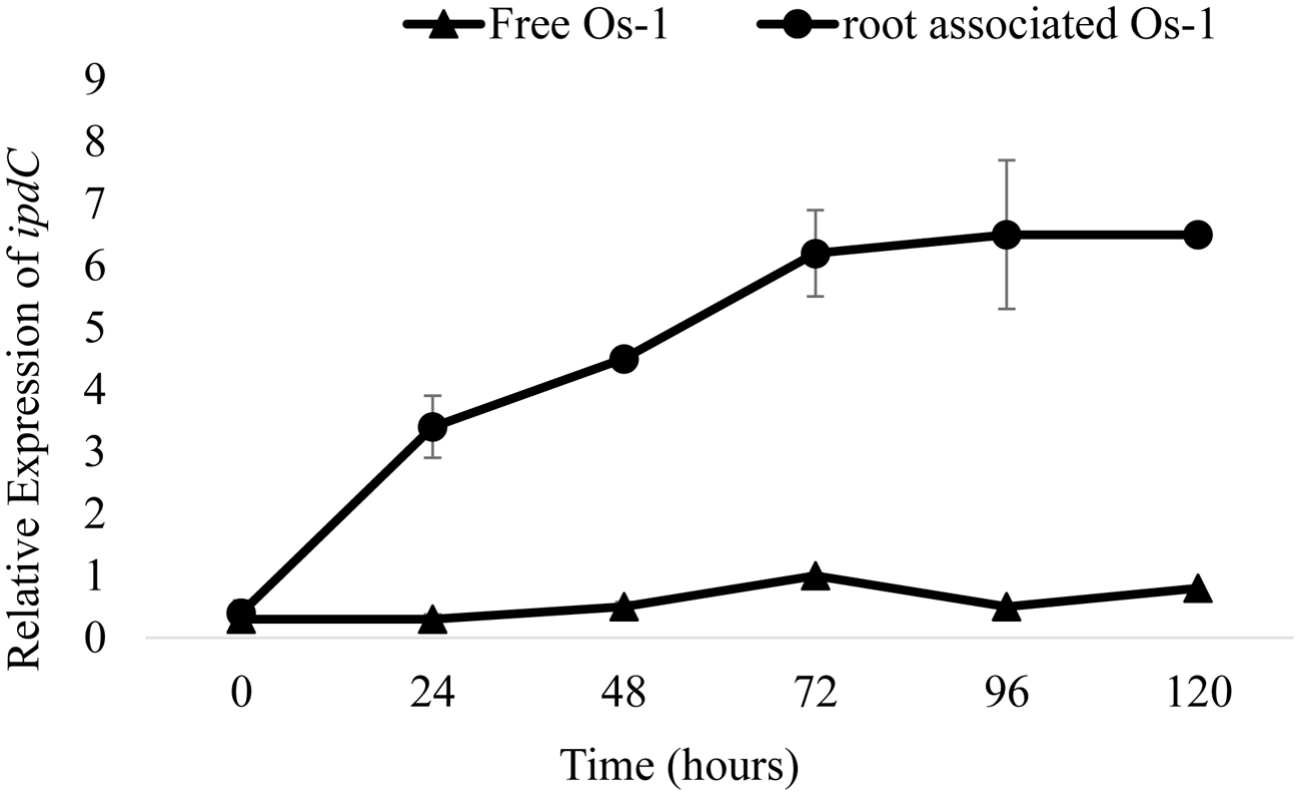
**Gene expression dynamics of putative *ipdC* genes of *Nostoc* in response to root colonization, based on qPCR of reverse-transcribed RNA extracts.** Relative expression of *ipdC* in *Nostoc* Os-1 grown alone (Free Os-1) or colonized on rice root (root associated Os-1). Graph represents data from three replicates in two independent experiments (means ± SD; *n* = 6).

To correlate the production of IAA with the expression of IAA biosynthesis genes, we first determine the expression of putative *ipdC* gene in free and rice roots associated *Nostoc* (grown under similar conditions) was measured by qPCR method and was compared. At time zero, expression of *ipdC* gene was similar in both root-associated and free living *Nostoc* (**Figure 1**). After 24 h, the *ipdC* expression was threefold greater in the root associated *Nostoc* sp. Os-1 compared to free grown Os-1 strain (**Figure 1**). In the following hours a fivefold increase in *ipdC* expression between root associated and free grown *Nostoc* sp. Os-1 was observed. The significant expression of putative *ipdC* gene in root-associated *Nostoc* strain led us to examine the involvement of this gene in root colonization by the cyanobacterium.

### GENERATION OF *ipdC* KNOCKOUT MUTANTS IN *Nostoc*

To investigate the biological function of IAA and potential involvement of *ipdC* in IAA production and root colonization, an *ipdC* knockout mutant was generated. A 169 bp of the coding region of the putative *ipdC* was replaced with a kanamycin resistance cassette to inactivate the gene. Since many copies of chromosomes can be found in a single cell of *Nostoc* (Jain et al., 2012), it was imperative to monitor the complete segregation of the wild allele by PCR. The knockout of the *ipdC* gene was confirmed by no amplification of *ipdC* specific primers by PCR. Using genomic DNA of *Nostoc* Os-1 (wild type strain), a fragment of 150 bp was amplified with the same pair of primers confirming the validity of the primers. Successful insertion with the right orientation of the fragment containing the sequence for resistance against kanamycin within chromosome of *ipdC* mutant strain (Δ*ipdC*), was confirmed by using kanamycin specific primers. The result of these two PCR reactions indicated removal of the functional *ipdC* gene in the mutant *Nostoc* strains.

### GROWTH ANALYSIS OF WILD TYPE, *ipdC* MUTANT, AND COMPLEMENTED *Nostoc* STRAINS

**FIGURE 2:**
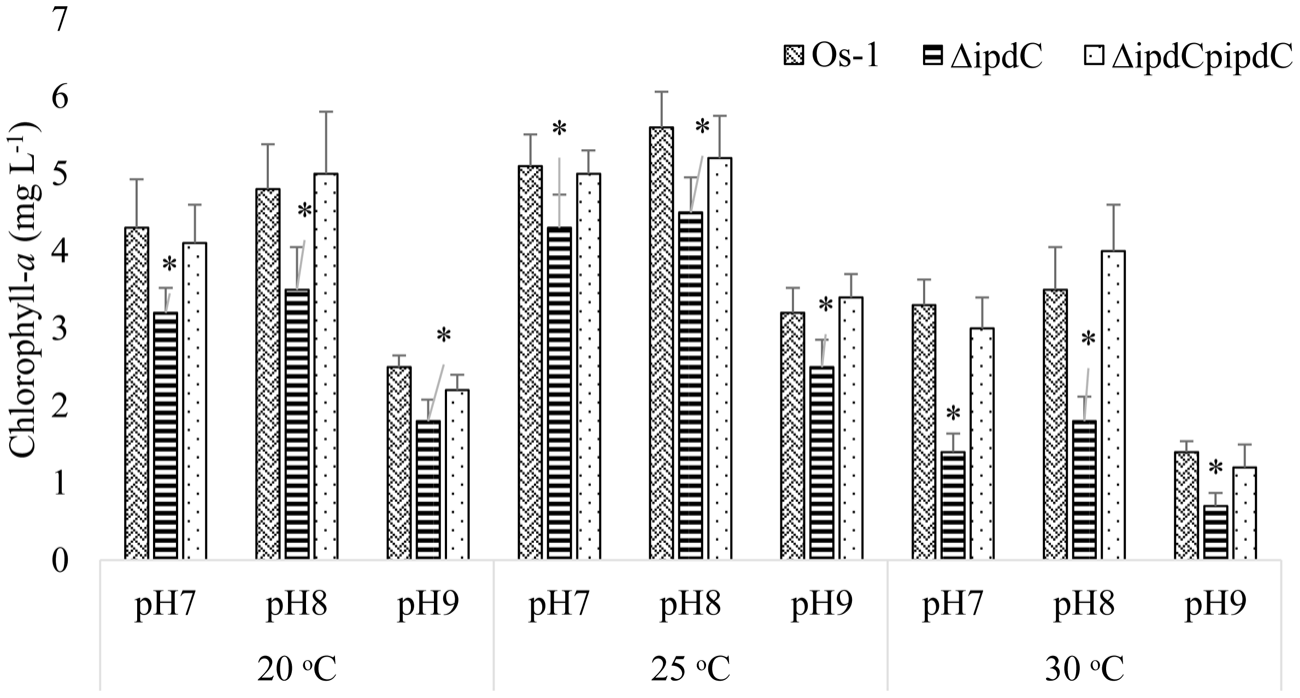
**Growth comparison for wild type (OS-1), *ipdC* knockout (Δ*ipdC*), and complementation (Δ*ipdC*p*ipdC*) strains of *Nostoc* cultured under different conditions of pH and temperature in BG11 media.** Growth is shown as chlorophyll-*a* concentration recorded after incubation of 15 days. Graph represents data from three replicates in two independent experiments (data are mean ± SD; *n* = 6). Asterics indicate a statistically significant difference (*p < 0.001; 3-way ANOVA) relative to wild and complementation strains.

**FIGURE 3:**
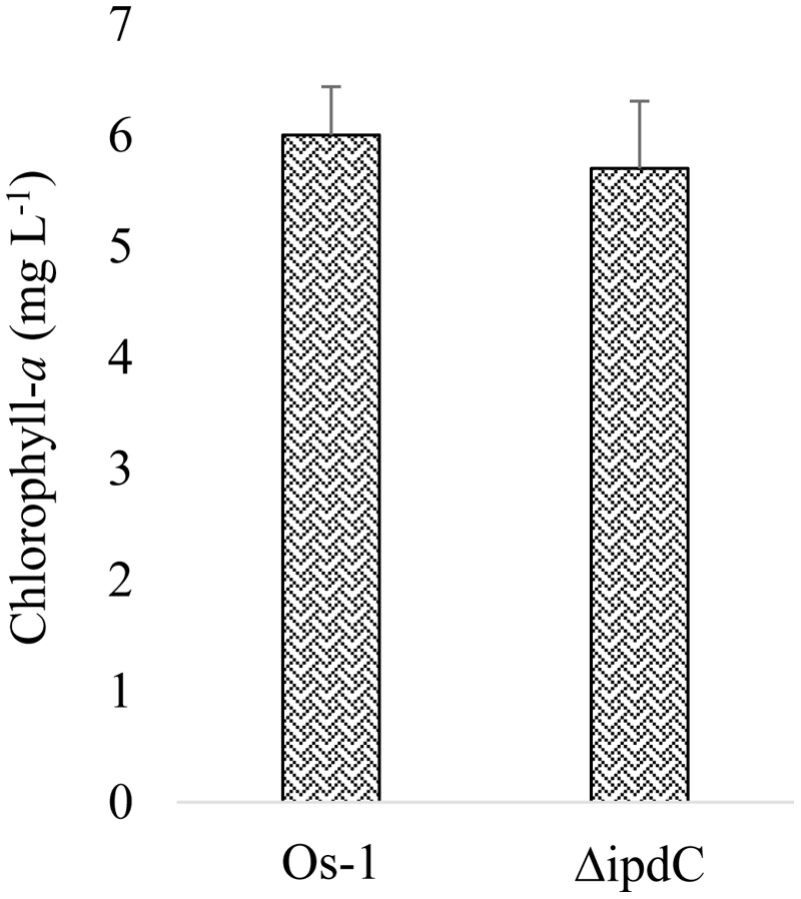
**Growth of wild type (Os-1) and mutant (Δ*ipdC*) *Nostoc* measured as chlorophyll-*a* in media containing roots inoculated with *Nostoc* strains.** Graph represent data from six replicates (mean ± SD). No significant difference was recorded (two samples *t-*test; *t* = 0.729)

To determine whether the deletion of *ipdC* affects *Nostoc* growth, we first analyze the growth of *Nostoc* by measuring chlorophyll-*a* content, which has been an acceptable method to quantify *Nostoc* growth and colonization in plant roots by Nilsson et al. (2002) and Hussain et al. (2013). Wild type (Os-1), *ipdC* knockout mutant (Δ*ipdC*), and complemented strain (Δ*ipdC*p*ipdC*) strains of *Nostoc* were cultured *in vitro* in BG11 medium at various pH and temperature. As shown in **Figure 2**, we found that the growth of wild type Os-1 was significantly greater than the growth of *ipdC* knockout mutant in all growth conditions tested (*p* < 0.05). Importantly, the complementation strain Δ*ipdC*p*ipd*C showed significant improvement in its growth (**Figure 2**). To determine if *Nostoc* growth was also compromised when it was grown in culture medium co-cultivated with rice seedlings in Petri plate, the *Nostoc* cells were collected and determined for the amount of chlorophyll-*a* after co-cultivated with rice seedlings for 15 days. There was no significant difference in the growth of wild type and Δ*ipdC* mutant when grown in this way (**Figure 3**).

### PHYTOHORMONES DETERMINATION

**FIGURE 4:**
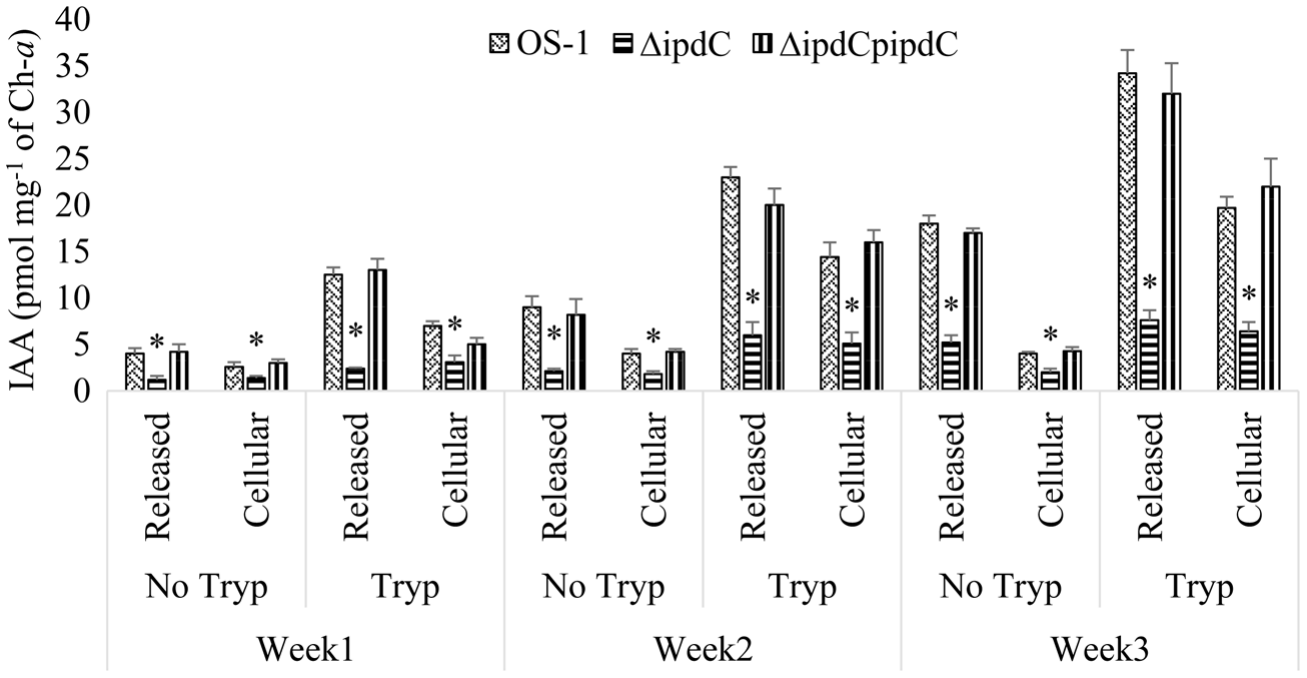
**Tryptophan dependent production and release of IAA by wild (Os-1), *ipdC* knockout mutant (Δ*ipdC*) and complementation strain (Δ*ipdC*p*ipdC*) of *Nostoc* over time.** IAA was determined in the culture filtrate and *Nostoc* filaments by UPLC-ESI-MS/MS after incubation of time indicated. Graph represents data from three replicates in two independent experiments (data are mean ± SD; *n* = 6). Asterisk indicate a statistically significant difference (*p < 0.001; 3-way ANOVA) relative to the respective means of wild type and complementation strains.

An optimum condition of 25°C, pH 8.0, and photoperiod of 8 light and 16 h dark were kept for growth and production of IAA by *Nostoc*. The wild-type strain of *Nostoc* released not only a significant amount of IAA in the culture media, but also retained IAA in the filaments under the set conditions. However, amount of IAA released was always greater than the amount of IAA retained in the filaments (**Figure 4**). The difference was increased sharply with increasing incubation time and reached to maximum (i.e., 78%) during the third week of incubation (**Figure 4**).

Also the amount of IAA peaked in culture incubated for 3 weeks in a medium with optimal amount of tryptophan (500 μg mL^-1^), higher amount of tryptophan was detrimental for the growth of *Nostoc*. After 3 weeks of incubation, a 78% reduction in the production of IAA (from 34.2 pmol mg^-1^ of ch-*a* to 7.6 pmol mg^-1^ of ch-*a*) was documented in the culture of mutant strain compared to wild type strain OS-1 (**Figure 4**). IAA to Biomass ratio in tryptophan supplemented media was also reduced by more than fourfold in Δ*ipdC* than that of wild type strain. The Δ*ipdC*p*ipdC* complementation strain restored wild-type level of IAA production. The results suggested that *ipdC* plays a role for IAA production, in which the amount was increased with time and in the presence of tryptophan (**Figure 4**).

### COLONIZATION OF *Nostoc* ON PLANT ROOTS

**FIGURE 5:**
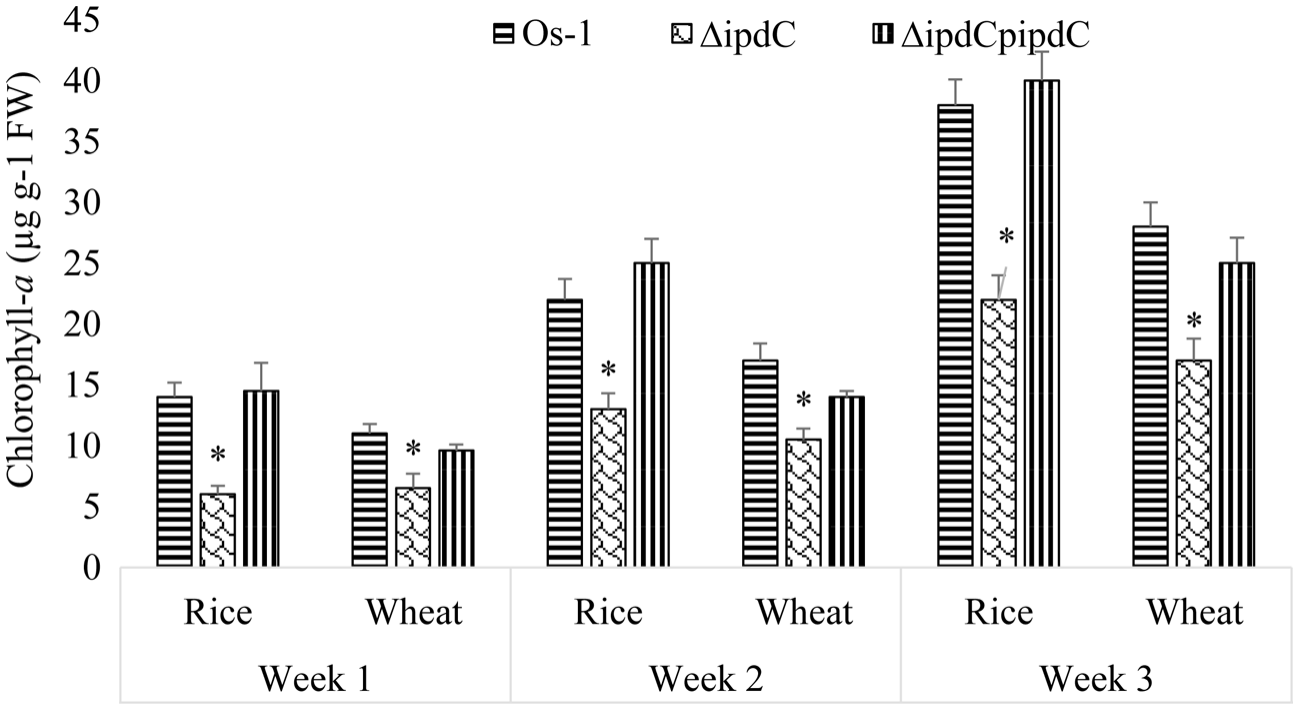
**Colonization of wild (Os-1), knockout (Δ*ipdC*) or complementation (Δ*ipdC*p*ipdC*) strain *Nostoc* on roots of rice and wheat roots over time.** Colonization was determined as chlorophyll-*a* (μg g^-1^ FW) in the roots. Graph represents data from three replicates in two independent experiments (data are mean ± SD; *n* = 6). Asterisk indicate a statistically significant difference (*p < 0.001; 3-way ANOVA) relative to wild and complementation strains.

Wild type strain of *Nostoc* efficiently colonized in the roots of rice as well as wheat seedlings. In the absence of *Nostoc*, no chlorophyll-*a* was detected in the roots of the selected plants. A gradual increase in the concentration of chlorophyll-*a* was recorded in the *Nostoc* colonized roots, as an indication of an increase in the growth of root-associated *Nostoc*. Concentration of chlorophyll-*a* in colonized root of rice and wheat by *Nostoc* Os-1 was increased from 14 to 12 μg g^-1^ FW (at the end of the first week) to 38 and 28 μg g^-1^ FW (by the end of the third week; **Figure 5**). We noticed that *ipdC* knockout strain Δ*ipdC* (low IAA mutant) was less efficient to colonize in the roots of rice as well as wheat seedlings, which is correlated with the reduced amounts of IAA (**Figure 5**). After co-cultivation of 1 week, significantly less amount (59% of the wild type strain) of Δ*ipdC* mutant was colonized in wheat root. By the end of second and third week, the amount of chlorophyll-*a* in the roots of wheat colonized by the mutant strain was 39 and 40% lower than that of the roots colonized by wild-type strain. For rice, the colonization efficiency Δ*ipdC* was only 43% of the wild-type strain of *Nostoc* during first week. In the subsequent weeks, the root chlorophyll-*a* was up to 40.9% lower in root colonized by the mutant as compared to the wild-type strain. The root colonization efficiency of *ipdC* knockout mutant was restored to wild type level by complementation (**Figure 5**).

### EXOGENOUS APPLICATION OF IAA RESTORED COLONIZATION EFFICIENCY OF THE Δ*ipdC*MUTANT

**FIGURE 6:**
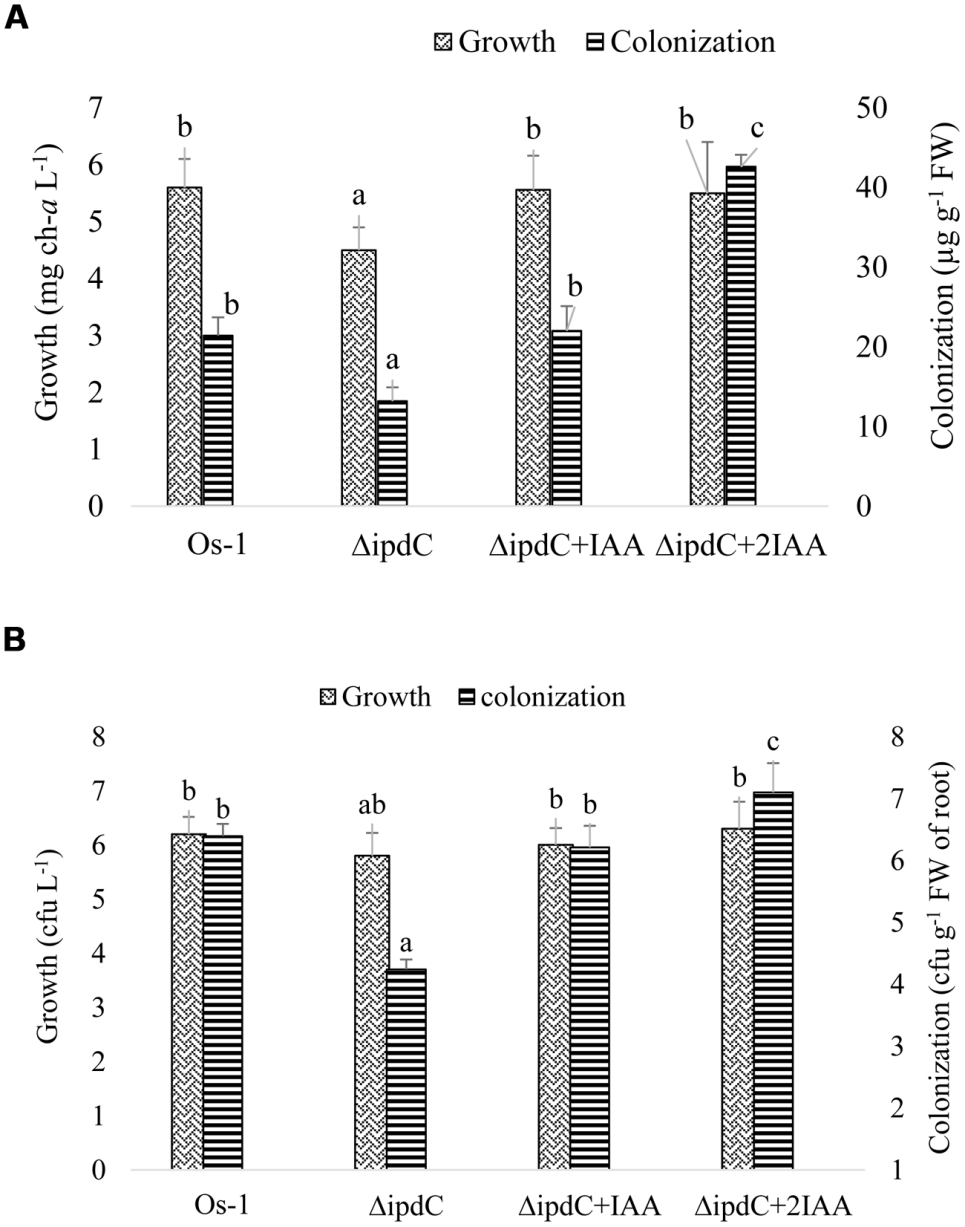
**Effect of exogenous IAA on the *in vitro* growth of free grown and root colonized *Nostoc*.** To reconstitute the concentration of IAA in mutant strain (Δ*ipdC*), IAA was supplemented to the culture media used to grow mutant *Nostoc* alone or in association with rice root in concentration exactly equal to (Δ*ipdC*+IAA) or double than (Δ*ipdC* +2IAA) the difference in its concentration produced by wild and mutant strains. Growth and colonization of *Nostoc* was determined by two methods, **(A)** quantification of chlorophyll-*a* and **(B)** counting *Nostoc* on agar plate (cfu). Graph represents data from nine replicates in two independent experiments (data are mean ± SD; *n* = 18). Similar bars labeled with different letters shows significant difference (*p* < 0.05; Duncan test).

When applied exogenously, IAA restored the *in vitro* growth as well as colonization efficiency of the mutant *Nostoc* when the concentration of chlorophyll-*a* was used as an indication of *Nostoc* growth (**Figure 6**). To confirm the correlation between chlorophyll-a concentration and plate counting method for cyanobacterial growth, colony-forming-unit (cfu) was also used to determine *Nostoc* growth. We showed that both chlorophyll-*a* and plate counting assays revealed the reduced root colonization efficiency of the Δ*ipdC* mutant strain (**Figure 6**). However, cfu of Δ*ipdC* was similar to that of wild type strain, which is inconsistent with the growth assay estimated by chlorophyll-*a* amounts.

### PHYTOSTIMULATION UNDER AXENIC CONDITIONS

We also determined the impacts of *ipdC* gene in plant growth. As shown in **Table 2**, seedlings of rice and wheat, inoculated with *Nostoc* sp. Os-1 (wild type strain) showed significantly greater growth than their respective controls. An overall increase of 23.5 and 50% in weight on dry and fresh weight basis was observed in wheat seedlings associated with *Nostoc* Os-1 as compared to the control. A similar response of rice to colonization by *Nostoc* was demonstrated. The effect of colonization on other growth parameters such as shoot length and root length of wheat and rice seedlings was remarkable. On the other hand, seedlings associated with mutant strain Δ*ipdC* grew similarly or only slightly better than their respective controls (no infection), suggesting the role of colonization and IAA in plant growth promotion by *Nostoc* (**Table 2**). However, complementation of wild-type *ipdC* restored the phytostimulatory efficiency of the knockout strain of *Nostoc*.

**Table 2:** Growth parameters of wheat and rice seedlings recorded after growth of 2 weeks under axenic conditions.

Strain	cm				mg plant^-1^			
	Shoot length		Root length		Fresh weight		Dry weight	
	Wheat	Rice	Wheat	Rice	Wheat	Rice	Wheat	Rice
Control	13.2 (a)	8.1 (a)	3.8 (a)	3.0 (a)	1.8 (a)	1.3 (a)	0.34 (a)	0.17 (a)
Os-1	16.4 (c)	11.0 (c)	6.5 (c)	5.4 (b)	2.7 (b)	1. 9 (b)	0.42 (b)	0.25 (b)
Δ*ipdC*	14.1 (b)	10.3 (b)	4.2 (b)	3.1 (a)	2.0 (a)	1.4 (a)	0.35 (a)	0.18 (a)
Δ*ipdC* p*ipdC*	15.5 (c)	12 (c)	5.8 (c)	4.9 (b)	2.8 (b)	2.0 (b)	0.36 (a)	0.23 (b)

*Seeds were primed with wild (Os-1), ipdC knockout (Δ*ipdC*), or complementation (ΔipdCpipdC) strains of Nostoc. Control seeds (cont.) were not primed with Nostoc. Different letters in the same column indicate significant difference between means (Duncan Multiple Range Test; *p* < 0.05).*

## DISCUSSION

Successful colonization on root is a key to plant growth promotion by cyanobacteria (Karthikeyan et al., 2007). Being renowned for its symbiotic association with plants, *Nostoc* was chosen to study their efficacy of colonization on plant roots as affected by self-produced IAA. A simple approach of chlorophyll-*a* determination in plant roots by Nilsson et al. (2002) and Hussain et al. (2013) was adopted to quantify root colonization by *Nostoc*. Under axenic conditions, the strains of *Nostoc* were able to produce IAA that enhanced their ability to colonize on wheat and rice roots. Also the induction of putative *ipdC* gene, a key gene of the IAA biosynthesis pathway from *Nostoc* (Sergeeva et al., 2002), suggested the involvement of IAA in the phenomenon, which is comparable to the findings of Tadra-Sfeir et al. (2011). It has been demonstrated that IAA production increases in *Rhizobium* when exposed to plant signals, the flavonoids, and IAA in turn enhances the release of flavonoids in root exudates of maize plant (Theunis et al., 2004; Dardanelli et al., 2008). To elaborate on the validity of the concept, *ipdC* gene was knocked out from *Nostoc* by homologous recombination. Significant loss in the ability of *Nostoc* to colonize on plant roots after knocking out *ipdC* provided an evidence of the role of IAA in plant-*Nostoc* interaction. IAA has been implicated in root colonization by rhizobacteria and fungi (Karabaghli-Degron et al., 1998; Lambrecht et al., 2000). Similarly, exogenous application of synthetic auxin, 2,4-dichlorophenoxyacetic acid (2,4-D), on wheat roots led to the formation of tumor-like structures, para-nodules, which were readily colonized by *Nostoc* (Gantar and Elhai, 1999). They had recorded up to 3.6 times more *Nostoc* on 2,4-D treated roots compared to untreated roots. Root colonization efficiency of IAA delivering non-heterocystous cyanobacteria was constantly more noteworthy than those that fail to offer this capacity (Ahmed et al., 2010). Besides 2,4-D, Cytokinins (Cks) are also used by *Nostoc* to colonize on roots (Hussain et al., 2013). It seems that auxin and Cks interact synergistically to promote root colonization by microbes. Synergistic interaction between Cks and auxin also exists in nodule organogenesis (Hwang et al., 2012). However, direct evidence of the involvement of *ipdC* or IAA in root colonization by *Nostoc* is reported for the first time. Our work was a validation of the previous speculations about the possible role of IAA in root colonization by cyanobacteria *Nostoc* (Nilsson et al., 2002). Indeed root colonization capability of *ipdC* knockout strain was reduced up to 41% and was not lost totally, showing the inclusion of elements other than IAA in this process. Furthermore, impact of knocking out *ipdC* from *Nostoc* was more intensive on its capacity to produce IAA as compared with its capability to colonize on plant root. Loss of *ipdC* gene from *Nostoc* was accompanied by a decline in its potential to promote plant development, consistent with previous conclusions that microbial-produced IAA has the potential to promote plant growth (Hussain and Hasnain, 2011). Reduction in the growth of *ipdC* knockout *Nostoc* was a validation of the previous study reporting the imperative part of IAA in cyanobacterial growth and development (Leganés et al., 1987).

Reduction in growth of *Nostoc* due to loss of *ipdC* gene suggested that this gene product was important for optimal growth of *Nostoc.* Thus, lower root colonization efficiency of the *ipdC* knockout mutant could be due to its compromised fitness rather than involving *ipdC* or IAA directly. Similar growth of wild type and *ΔipdC Nostoc* in culture medium co-cultivated with plant roots suggested the role of IAA in root colonization. Additionally, the evidence that the *in vitro* growth, root colonization efficiency, and plant growth promoting activity of the *ipdC* mutant strain could be restored when IAA was exogenously applied in the culture medium also suggested the involvement of IAA in root colonization and plant promoting efficiency of *Nostoc*.

Though chlorophyll-*a* determination is an acceptable method used by Nilsson et al. (2002) and Hussain et al. (2013) to quantify root colonization by *Nostoc*, chlorophyll-*a* amounts may be an indicative of the overall cell growth instead of the number of cyanobacterial cells or filaments. Thus, colony forming units (cfu) of free grown and root-associated *Nostoc* were counted on agar plate. While both chlorophyll-*a* and plate counting assays revealed the reduced root colonization efficiency of the Δ*ipdC* mutant strain, reduced chlorophyll-*a* in *ΔipdC* during *in vitro* growth was not confirmed by plate counting analysis. These results indicated that chlorophyll-*a* concentration is not necessarily correlated with cyanobacterial growth. These results suggested that the reduction of chlorophyll-a in the *in vitro* grown mutant strain was not due to the reduced growth but was due to reduction in chlorophyll-a synthesis/accumulation. If so, it is plausible to suggest a role of IAA in chlorophyll-a synthesis by *Nostoc*. Stimulatory effect of IAA on chlorophyll-*a* and growth of *Spirulina* has been observed (Mohammed and Mohd, 2011). Another possibility is that IAA might affect hormogonia production (Hunter and Provasoli, 1964), which is necessary for root colonization as well as growth of cyanobacteria. Also, plate counting may result in over estimation of *Nostoc* due to filament breakage or hormogonia formation. Similarly, incomplete separation of the filaments in clusters may lead to under estimation of cell number (Whitton, 2002). To this end, we could not exclude the possibility that the compromised growth of Δ*ipdC* mutant may indirectly lead to its reduced colonization on plant root. However, the induction of *ipdC* gene expression upon its association with roots and the correlation of IAA production with root colonization efficiency and plant growth promoting activity indeed supported a role of IAA in root colonization by *Nostoc*.

Our findings highlighted the important role of IAA in root colonization by *Nostoc*. Restoration of root colonization ability of an *ipdC* mutant strain by exogenous IAA addition further confirmed the role of IAA in plant-*Nostoc* interaction. It may be concluded that IAA plays a vital role in root colonization by *Nostoc* and consequently plant growth promotion. Additionally, *Nostoc*’s own growth and development may be affected by IAA.

## Conflict of Interest Statement

The authors declare that the research was conducted in the absence of any commercial or financial relationships that could be construed as a potential conflict of interest.
